# A retrospective tomotherapy image‐guidance study: analysis of more than 9,000 MVCT scans for ten different tumor sites

**DOI:** 10.1120/jacmp.v15i6.4663

**Published:** 2014-11-08

**Authors:** Patricia Sánchez‐Rubio, Ruth Rodríguez‐Romero, Pablo Castro‐Tejero

**Affiliations:** ^1^ Department of Medical Physics Hospital Universitario Puerta de Hierro Majadahonda Madrid Spain

**Keywords:** tomotherapy, MVCT, image guidance, on‐line and off‐line protocols

## Abstract

The purpose of this study was to quantify the systematic and random errors for various disease sites when daily MVCT scans are acquired, and to analyze alternative off‐line verification protocols (OVP) with respect to the patient setup accuracy achieved. Alignment data from 389 patients (9,418 fractions) treated at ten different anatomic sites with daily image‐guidance (IG) on a helical tomotherapy unit were analyzed. Moreover, six OVP were retrospectively evaluated. For each OVP, the frequency of the residual setup errors and additional margins required were calculated for the treatment sessions without image guidance. The magnitude of the three‐dimensional vector displacement and its frequency were evaluated for all OVP. From daily IG, the main global systematic error was in the vertical direction (4.4–9.4 mm), and all rotations were negligible (less than 0.5°) for all anatomic sites. The lowest systematic and random errors were found for H&N and brain patients. All OVP were effective in reducing the mean systematic error to less than 1 mm and 0.2° in all directions and roll corrections for almost all treatment sites. The treatment margins needed to adapt the residual errors should be increased by 2–5 mm for brain and H&N, around 8 mm in the vertical direction for the other anatomic sites, and up to 19 mm in the longitudinal direction for abdomen patients. Almost 70% of the sessions presented a setup error of 3 mm for OVPs with an imaging frequency above 50%. Only for brain patients it would be feasible to apply an OVP because the residual setup error could be compensated for with a slight margin increase. However, daily imaging should be used for anatomic sites of difficult immobilization and/or large interfraction movement.

PACS numbers: 87.55.‐x, 87.56.‐v

## I. INTRODUCTION

In the last few years, 3D volumetric imaging, such as megavoltage or kilovoltage cone‐beam or fan‐beam CTs, have become popular image‐guided radiotherapy (IGRT) solutions^(1)^ because they provide more anatomical information than electronic portal imaging. Helical tomotherapy is an integrated system which offers the possibility of delivering helical IMRT and fan‐beam megavoltage computed tomography (MVCT) imaging,^(2)^ and it can be used to treat a wide variety of disease sites such as head and neck, brain, pelvis, lung, and breast. Moreover, advantages have been reported for large volume treatments.^3^, ^4^

The high conformal plans with steep dose gradients and the decrease in geometrical uncertainty in the patient setup due to IGRT have made it possible to consider margin reduction. The strategies to eliminate the setup error are related with image‐guidance frequency (IGf): on‐line verification protocol (100% IG) allows setup corrections on a daily basis and, therefore, systematic and random errors are corrected; for off‐line verification protocols (OVP) where images are not acquired in all treatment fractions, only the elimination of systematic errors is achieved. However, the margins cannot be reduced to zero because there are systematic errors that remain, despite the use of IGRT, such as errors in target and organs delineation, imaging acquisition and registration technique accuracy, or random errors such as tumor motion during the course of radiotherapy treatment.

Although it is clear that the use of an accurate and precise image‐guidance technique results in the best patient positioning over the course of treatment, the cost associated with imaging has to be considered, in terms of the additional patient‐absorbed dose^5^, ^6^, ^7^ and/or machine time.

The purpose of this study is to quantify the systematic and random errors that are obtained for various disease sites when daily MVCT scans are acquired and to analyze alternative OVP related to the patient setup accuracy achieved, and to determine how these protocols impact patients' treatments.

## II. MATERIALS AND METHODS

Positioning corrections and different image guidance protocols were analyzed for more than 350 patients treated with a helical tomotherapy unit (TomoTherapy Hi·Art II, Accuray Inc., Sunnyvale, CA). IEC‐61217 conventions^(8)^ have been followed to express translation and rotation data.

### A. Patient setup and image acquisition

Patients were grouped by anatomic treatment sites and the kind of immobilization used. Those treatments under 5 fractions were not used in this analysis. So, positioning corrections were analyzed for 40 brain (730 fractions), 112 head and neck (H&N) (3,489 fractions), 15 esophagus (413 fractions), 24 lung (474 fractions), 19 breast (491 fractions), 20 chest (292 fractions), 41 prostate (1,508 fractions), 37 pelvis (888 fractions), 27 abdomen (623 fractions), and 15 extremity (303 fractions) patients.

Patients were positioned by aligning skin tattoos or markers on mask with the treatment room's red lasers and immobilized with the following devices: the Posicast Thermoplastics head and shoulder masks (CIVCO Medical System, Kalona, IA) for H&N and esophagus; BrainLAB Mask System (BrainLAB AG, Feldkirchen, Germany) for brain; a wingboard for lung and also for those chest patients whose tumor was in the upper chest (Hodgkin lymphoma and dorsal mestastasis); the Posiboard‐2 breastboard (CIVCO Medical Solution) for breast; a CombiFix (Feetfix and Kneefix cushions) for abdomen (pancreas, stomachs, retro peritoneum) and the pelvic location (prostate, bladder, rectum and gynecological patients); and thermoplastic masks or vacuum cushions (Posicast Thermoplastics and Vac‐Lok Cushions from CIVCO Medical Solutions, respectively) for extremities.

In our current clinical practice, a MVCT scan is obtained for all patients before each fraction. The slice thickness is chosen based on the size of the structures needed for image registration and therefore trying not to have an excessively long scan time. In general, normal slice thickness (4 mm) was used, except for some cases of brain (2 mm) and extremity patients (6 mm). The scan length in the superior–inferior direction included the entire planning target volume (PTV) whenever possible. Then, the MVCT to kilovoltage CT (kVCT) image registration was performed by the TomoTherapy system software to determine translational and rotational (roll, pitch, and yaw)^(9)^ corrections. Usually, image registration was based on bony or bony and soft tissue anatomy. The accuracy and precision of the MVCT‐kVCT registration is lower than 1 mm, as has been proved by other authors.^10^, ^11^ As pitch and yaw deviations cannot be corrected by gantry or automatic couch offsets, the patient was repositioned if these deviations were greater than 3°. Otherwise, the proposed automatic pitch and yaw corrections were set at 0°, and the therapist executed additional manual shifts to compensate for them and get a fine‐tuned registration by comparing the bony landmarks and other anatomical structures near the tumor localization (i.e., upper cervical spine and/or base of skull was used for H&N or dorsal spine, ribs or the tumor itself for lung patients); whereas the prostate anatomy was employed to prostate patients, at the same time that bladder and rectum filling were checked. If proper alignment of all landmarks was not possible, the closest landmarks to the tumor volume were given priority, along with the isodose distribution overlaid onto the MVCT images, so that both target coverage and dose to the critical structures (OAR) can be checked.

### B. Image‐guidance protocols

In order to determine the magnitude of the residual alignment errors and the feasibility of implementing an off‐line verification protocol^12^, ^13^ to reduce the imaging workload, several OVP were simulated retrospectively using the daily positioning data.

#### B.1 OVPNonimaging (NI)

No MVCT would be obtained. The patient would be positioned based on the alignment of the skin tattoos or markers. The systematic drop in the couch height was not considered.

#### B.2 OVPFirst five fractions (FFF)

MVCT scans would be acquired during the first 5 fractions. Mean shifts were calculated from these data and were applied for the subsequent treatments without imaging. This is similar to the nonaction level (NAL) protocol described by de Boer et al.^12^, ^13^, ^14^

#### B.3 OVPweekly imaging with 3 mm action level (W3mmAL)

The first fraction's initial correction would be updated when weekly MVCT showed shifts ≥3mm, simulating common clinical practice of weekly portal imaging.

#### B.4 OVPFFF plus weekly imaging with patient‐specific action level (FFF+WpatAL)

The same as for the OVP FFF, but MVCT pretreatment would be performed every 5 fractions. In the fractions without IG, the mean shift calculated from the corrections of the first 5 fractions would be applied, unless any subsequent patient's shift would be greater than twice standard deviation of the shifts from the first five days. In this last case, the following fractions would be corrected by the new systematic shift. This protocol is similar to the extended nonaction level proposed by de Boer and Heijmen.^(14)^

#### B.5 OVPAlternate day with a running mean (AD)

The patient would undergo imaging for odd fractions until the end of the treatment course. Even fractions would be corrected determining the running average of odd fractions.

#### B.6 OVPAlternate week (AW)

MVCTs would be performed on odd weeks considering weeks as 5 consecutive fractions. The corrections applied to fractions of even weeks would be based on the calculated mean shifts of previous week

To determinate the workload of each protocol, the percentage of sessions that would have been imaged was scored. For an individual patient, IGf was calculated as the ratio of IG sessions to the total number of treatment fractions; whereas, for the OVPs, it was calculated as IGf average for each location.

### C. Data analysis

Firstly, the setup correction data obtained from the daily image‐guided sessions were examined. Population‐based parameters such as mean systematic error, M(μ), its standard deviation, Σ(μ), and the average random (RMS(σ)) error were determined based on the methodology introduced by van Herk^(15)^ for each anatomical treatment site.^16^, ^17^, ^18^

Secondly, the residual setup errors were calculated as the difference between the daily alignment and the resultant alignment if a particular OVP had been followed. The frequency of residual setup errors was studied for fractions without IG to test the predictive quality of each scenario.

In addition, CTV‐to‐PTV margins were calculated according to van Herk's formula.^15^, ^19^ These calculated margins should not be regarded as clinical margins because the systematic errors caused by the delineation process and imaging/registration accuracy have not been taken into account. They should be considered as additional minimal setup margins to guarantee the correct target dosage. In the case of the different OVP, the calculated margins should be understood as the treatment margins that would be required on days without IG to accommodate the residual errors.

## III. RESULTS

### A. Daily setup correction

**Figure 1:**
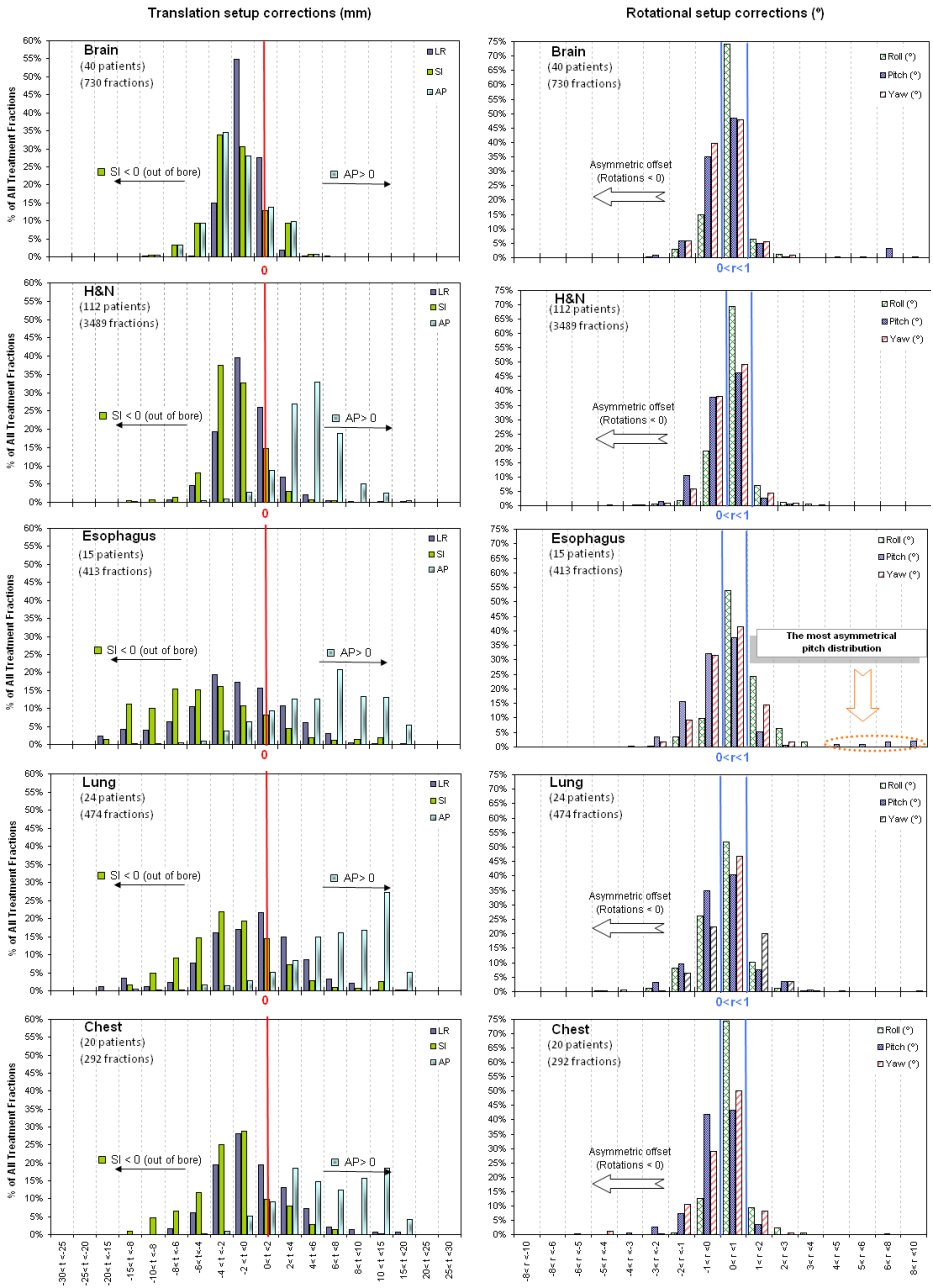
Histograms of translational (truncated at 30 mm) and rotational setup corrections for different anatomic treatment sites.

**Figure 2:**
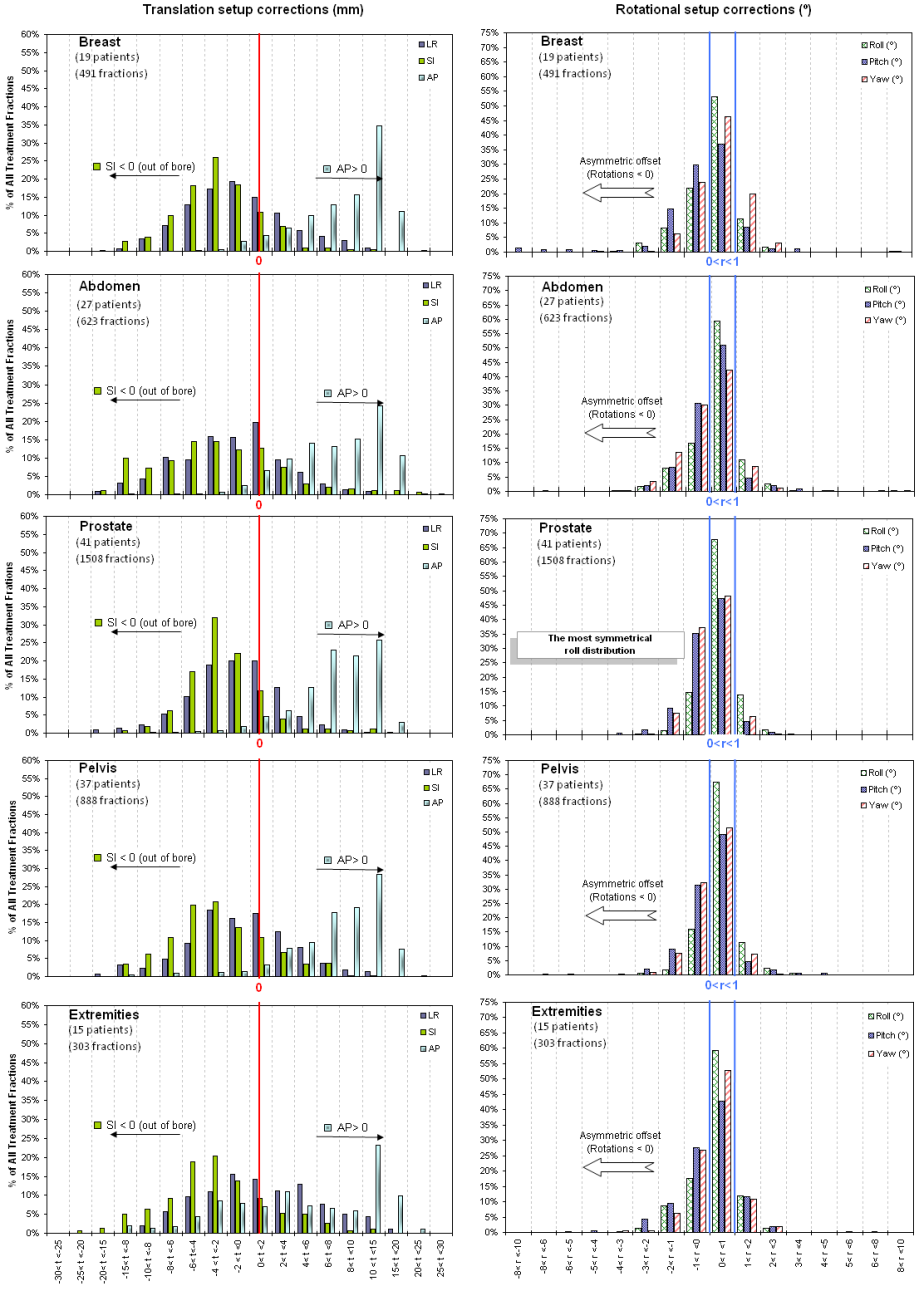
Histograms of translational (truncated at 30 mm) and rotational setup corrections for different anatomic treatment sites.

Histograms of translational and rotational setup corrections display the ranges of corrections made for each location (Figs. 1 and 2). A positive systematic error was observed from vertical (AP) distribution for all anatomic treatment sites. Longitudinal (SI) corrections were higher than lateral (LR) corrections, and always out of the bore. LR and SI distribution for H&N and brain locations are sharply peaked about −2mm, whereas prostate, breast, and chest distributions are slightly wider. Esophagus, lung, pelvis, abdomen, and extremities are the widest of all distributions.

Rotational distributions are similar between the different treatment sites and are centered around 0°–1°, and show a slightly asymmetric offset towards negative rotational corrections, except for pitch distribution for esophagus patients.

**Figure 3:**
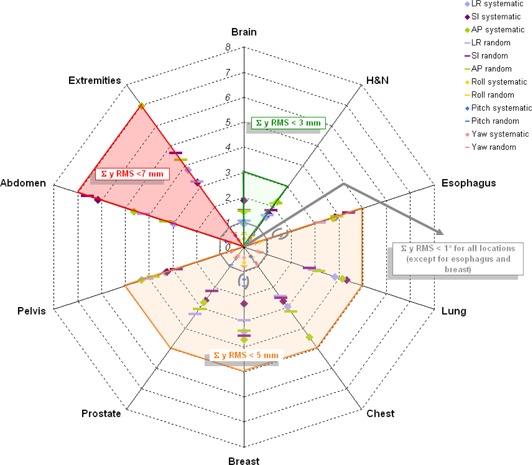
Variation in in Σ(μ) and RMS(σ) for translations and rotations by treatment site. (Scale in millimetres (mm) for translations or degrees (°) for rotations).

Variations in Σ(μ) and RMS(σ) are displayed in Fig. 3. For H&N and brain patients, both are lower than for the others treatment sites (1–2mm) due to the good immobilization device used. On the contrary, abdomen and extremity patients have systematic errors of 6.14 mm and 6.97 mm in SI and AP direction, respectively. The abdomen patients' group included gastric, suprarenal, retroperitoneum, and pancreatic cases, the latter being those which have the highest systematic and random errors: 5.22 mm and 10.64 mm. Note, too, the pitch errors of 1.53° and 1.30° for esophagus and breast patients.

### B. Off‐line protocols

Tables 1, 2 show all protocols are effective in reducing M(μ) under 1 mm and 0.2° in all directions and roll corrections, respectively, excluding esophagus and abdomen, with maximum M(μ) values of −2mm in SI direction, −1.5mm in AP direction for extremity, and −0.4° roll for breast patients. Σ decreases by increasing imaging frequency, whereas the RMS(σ) is not affected. The average random residual error is lower than 2 mm for brain and H&N patients. For esophagus, prostate, pelvis, breast, lung, and chest patients it remains around 3 or 4 mm, possibly due to interfraction movement, whereas for abdomen and extremity patients is even higher, up to 5 or 7 mm, due not only to the interfraction movement, but also to the difficulty of achieving a good registration for long PTVs. For roll correction, the random component does not change with any of the imaging protocols (0.5°‐0.9° for all treatment sites).

**Table 1:** Results of translational setup error analysis from daily image‐guided and for each off‐line verification protocol (for the fractions without image guidance).

			*LR (mm)*				*SI (mm)*				*AP (mm)*			
	*Protocol*	*IGf (%)*	*M(μ)*	σ	*RMS(σ)*	*CTV‐PTV Margin*	*M(μ)*	σ	*RMS(σ)*	*CTV‐PTV Margin*	*M(μ)*	σ	*RMS(σ)*	*CTV‐PTV Margin*
*BRAIN (40 patients) (370 fractions)*	NI	0%	−0.6	0.9	1.0	3	−1.5	1.9	1.5	6	4.4	1.4	1.4	5
	FFF	47%	−0.2	0.7	0.9	2	0.1	1.1	1.3	4	−0.1	0.9	1.7	3
	W3mmAL	22%	0.0	0.7	1.1	3	0.3	1.3	1.6	4	0.0	0.7	1.7	3
	FFF+WpatAL	56%	−0.1	0.6	1.0	2	0.4	0.9	1.4	3	−0.2	0.5	2.0	3
	AD	53%	−0.1	0.5	0.9	2	0.2	0.9	1.7	3	0.0	0.7	1.0	3
	AW	66%	−0.1	0.6	1.0	2	0.2	0.9	1.3	3	0.0	0.6	2.1	3
*H&N (112 patients) (3489 fractions)*	NI	0%	−0.7	1.5	1.6	5	−1.7	1.7	1.8	5	4.5	2.2	2.1	7
	FFF	17%	0.1	1.1	1.6	4	0.0	1.5	1.8	5	−0.4	1.7	2.1	6
	W3mmAL	21%	0.2	1.2	1.6	4	0.1	1.5	2.1	5	−0.1	1.3	2.5	5
	FFF+WpatAL	33%	0.1	0.9	*1.6*	4	0.0	1.4	2.0	5	−0.1	1.3	2.4	5
	AD	51%	0.1	0.6	1.6	3	0.0	0.9	1.8	3	0.1	1.0	2.1	4
	AW	54%	0.0	0.6	1.6	3	0.1	0.7	1.8	3	−0.1	0.8	2.2	3
*ESOPHAGUS (15 patients) (413 fractions)*	NI	0%	−1.8	3.9	3.2	12	−4.0	3.8	4.4	13	5.8	3.7	3.9	12
	FFF	18%	−0.3	2.1	3.2	8	−1.6	3.7	4.0	12	−1.0	3.1	3.9	10
	W3mmAL	21%	0.2	1.2	1.6	4	0.1	1.5	2.1	5	−0.1	1.3	2.5	5
	FFF+WpatAL	33%	0.2	2.1	3.6	8	−1.3	3.6	4.2	12	−0.6	3.6	5.2	13
	AD	50%	−0.5	1.4	3.2	6	0.3	1.7	5.0	8	−0.6	1.9	3.4	7
	AW	53%	−0.3	1.1	3.4	5	−0.3	1.6	5.3	8	0.0	2.1	3.5	8
*LUNG (24 patients) (474 fractions)*	NI	0%	−0.6	3.8	3.0	12	−1.7	4.3	2.7	13	7.0	4.1	3.1	13
	FFF	33%	0.3	2.0	2.9	7	−0.3	2.3	2.8	8	−0.8	2.7	2.9	9
	W3mmAL	21%	0.0	2.5	3.3	9	−0.2	1.7	4.0	7	−0.2	2.5	4.5	9
	FFF+WpatAL	46%	−0.1	1.6	3.2	6	−0.2	1.8	2.9	6	−0.6	2.1	3.4	8
	AD	51%	−0.1	1.9	3.2	7	−0.3	1.5	3.0	6	−0.3	1.8	3.2	7
	AW	54%	0.5	1.8	3.0	7	−0.3	1.8	2.8	6	−0.4	1.9	2.9	7
*CHEST (20 patients) (292 fractions)*	NI	0%	0.3	3.0	2.4	9	−2.1	2.7	2.5	8	4.7	4.5	3.2	13
	FFF	50%	0.6	1.9	3.0	7	0.1	1.6	2.9	6	−0.5	2.2	3.1	8
	W3mmAL	34%	−0.2	1.8	3.1	7	0.4	2.2	3.0	8	0.4	2.7	4.4	10
	FFF+WpatAL	59%	0.6	1.8	3.1	7	0.0	1.3	3.2	6	−0.6	1.3	3.1	5
	AD	60%	−0.3	1.1	2.3	4	0.8	1.9	2.5	6	0.0	2.7	3.8	9
	AW	70%	0.4	1.8	3.0	7	−0.2	1.5	2.8	6	−0.4	1.7	3.0	6
*BREAST (19 patients) (491 fractions)*	NI	0%	−0.9	3.4	2.9	11	−2.9	2.3	3.6	8	9.4	3.7	3.4	12
	FFF	20%	−0.8	1.7	2.8	6	−0.3	1.6	3.6	7	0.1	1.8	3.3	7
	W3mmAL	21%	−0.5	2.1	3.6	8	−0.2	1.6	4.5	7	0.0	1.8	4.3	8
	FFF+WpatAL	34%	−0.7	1.7	2.7	6	−0.1	1.3	3.9	6	−0.3	1.4	4.1	6
	AD	51%	0.0	1.3	3.1	5	0.0	1.2	4.0	6	0.4	1.9	3.4	7
	AW	56%	−0.6	0.9	2.9	4	−0.2	1.2	4.0	6	−0.3	1.6	3.1	6
*ABDOMEN (27 patients) (623 fractions)*	NI	0%	−1.7	3.0	4.2	10	−2.1	6.1	6.6	20	8.2	4.6	3.3	14
	FFF	29%	0.0	2.2	4.4	9	−2.0	5.7	6.5	19	0.1	1.6	3.1	6
	W3mmAL	21%	−1.0	3.5	4.9	12	−0.1	4.1	7.7	16	0.4	2.5	4.7	9
	FFF+WpatAL	41%	0.0	2.2	4.9	9	−1.9	5.7	6.9	19	−0.1	1.4	3.5	6
	AD	51%	−0.8	2.6	4.3	9	−0.8	4.4	7.4	16	0.5	1.8	3.8	7
	AW	58%	−0.3	1.9	4.5	8	−1.1	3.8	7.8	15	0.0	1.3	3.5	6
*PROSTATE (41 patients) (1508 fractions)*	NI	0%	−1.1	2.6	3.3	9	−2.1	2.6	2.2	8	7.8	2.7	3.0	9
FFF	14%	0.1	2.1	3.4	7	0.0	1.3	2.1	5	−0.3	2.2	3.0	8
W3mmAL	21%	0.3	1.6	3.9	7	0.5	1.6	2.7	6	−0.3	1.6	4.2	7
FFF+WpatAL	30%	−0.1	1.3	4.2	6	0.1	1.0	2.4	4	−0.1	1.7	3.7	7
AD	50%	0.2	1.7	3.5	7	−0.2	0.9	2.3	4	−0.2	1.5	3.2	6
AW	52%	0.3	2.0	3.7	8	0.1	0.7	2.2	3	0.1	1.2	3.1	5
*PELVIS (37 patients) (888 fractions)*	NI	0%	−0.7	3.3	4.1	11	−2.7	3.2	2.8	10	8.3	4.3	3.8	13
	FFF	26%	−0.6	2.3	4.0	8	−0.2	2.0	2.8	7	−0.3	2.6	3.8	9
	W3mmAL	21%	−0.2	3.3	4.9	12	−0.2	1.9	3.3	7	0.0	1.9	4.9	8
	FFF+WpatAL	39%	−0.4	2.1	4.1	8	−0.2	1.6	3.0	6	−0.3	2.4	4.1	9
	AD	51%	0.2	3.0	3.7	10	−0.3	1.2	3.0	5	0.5	2.0	4.0	8
	AW	56%	−0.5	2.1	4.2	8	−0.2	1.6	3.0	6	−0.3	2.0	3.9	8
*EXTREMITIES (15 patients) (303 fractions)*	NI	0%	0.6	3.8	3.2	12	−3.3	3.2	4.6	11	4.4	7.0	4.3	20
	FFF	40%	−0.4	3.2	*3.3*	10	−0.6	4.5	4.4	14	−1.5	2.8	4.7	10
	W3mmAL	22%	0.0	1.6	*3.9*	7	−1.0	4.4	5.1	15	0.6	3.1	5.7	12
	FFF+WpatAL	51%	−0.5	2.9	3.4	10	−1.0	4.6	5.1	15	−1.2	2.6	4.9	10
	AD	53%	−0.5	1.4	3.2	6	−0.8	3.7	5.3	13	0.4	2.0	4.8	8
	AW	64%	−0.5	2.7	3.3	9	−0.7	3.9	4.9	13	−0.7	1.9	5.6	9

**Table 2:** Results of rotational setup error analysis from daily image‐guided and for each off‐line verification protocol (for the fractions without image guidance).

			*Roll (°)*			*Pitch (°)*			*Yaw (°)*		
	*Protocol*	*IGf (%)*	*M(*μ*)*	Σ	*RMS(*σ*)*	*M(*μ*)*	Σ	*RMS(*σ*)*	*M(*μ*)*	Σ	*RMS(*σ*)*
*BRAIN (40 patients) (370 fractions)*	NI	0%	0.2	0.3	0.5	0.1	1.1	0.6	0.0	0.3	0.6
	FFF	47%	0.0	0.4	0.5	0.0	0.5	0.6	0.1	0.3	0.5
	W3mmAL	22%	0.0	0.7	0.6	0.0	0.6	0.6	−0.2	0.7	0.5
	FFF+WpatAL	56%	0.0	0.3	0.5	0.0	0.4	0.6	0.0	0.3	0.5
	AD	53%	0.0	0.4	0.5	0.0	0.3	0.6	0.0	0.6	0.6
	AW	66%	0.0	0.3	0.6	−0.1	0.3	0.6	0.0	0.3	0.6
H&N (112 patients) (3489 fractions)	NI	0%	0.2	0.4	0.5	−0.2	0.4	0.6	−0.1	0.4	0.6
	FFF	17%	0.0	0.3	0.5	0.0	0.4	0.6	0.1	0.4	0.6
	W3mmAL	21%	0.0	0.5	0.5	0.0	0.5	0.6	0.1	0.6	0.6
	FFF+WpatAL	33%	0.0	0.3	0.5	0.0	0.4	0.6	0.1	0.4	0.7
	AD	51%	0.0	0.2	0.6	0.0	0.2	0.6	0.0	0.3	0.7
	AW	54%	0.0	0.2	0.6	0.0	0.2	0.6	0.0	0.2	0.7
*ESOPHAGUS (15 patientes) (413 fractions)*	NI	0%	0.6	0.6	0.6	0.1	1.5	1.1	0.1	0.5	0.7
	FFF	18%	0.1	0.5	0.7	−0.4	0.6	1.1	−0.1	0.4	0.7
	W3mmAL	21%	0.0	0.5	0.5	−0.2	0.7	1.2	−0.1	0.9	0.7
	FFF+WpatAL	33%	0.0	0.4	0.7	−0.3	0.5	1.1	−0.1	0.3	0.8
	AD	50%	−0.1	0.3	0.7	−0.1	0.2	0.9	−0.1	0.4	0.8
	AW	53%	0.0	0.2	0.7	−0.1	0.3	1.0	−0.1	0.2	0.8
*LUNG (24 patients) (474 fractions)*	NI	0%	0.1	0.6	0.7	−0.1	0.6	0.7	0.3	0.6	0.8
	FFF	33%	0.0	0.4	0.7	−0.1	0.6	0.7	−0.1	0.5	0.6
	W3mmAL	21%	−0.1	0.7	0.7	−0.1	0.4	0.7	0.1	0.9	0.8
	FFF+WpatAL	46%	0.0	0.4	0.7	0.0	0.5	0.7	−0.2	0.5	0.7
	AD	51%	−0.1	0.4	0.7	0.1	0.3	0.7	−0.1	0.5	0.8
	AW	54%	0.0	0.3	0.6	0.0	0.4	0.7	−0.1	0.4	0.7
*CHEST (20 patients) (292 fractions)*	NI	0%	0.4	0.4	0.5	−0.2	0.4	0.8	0.0	0.9	0.7
	FFF	50%	−0.1	0.3	0.5	−0.3	0.6	0.7	−0.2	0.4	0.6
	W3mmAL	34%	−0.2	0.6	0.5	−0.1	0.4	0.8	0.1	0.6	0.8
	FFF+WpatAL	59%	0.0	0.3	0.6	−0.3	0.5	0.8	−0.2	0.4	0.6
	AD	60%	−0.1	0.4	0.6	0.0	0.4	0.8	0.2	0.6	0.8
	AW	70%	0.0	0.2	0.5	−0.2	0.3	0.8	−0.1	0.3	0.6
*BREAST (19 patients) (491 fractions)*	NI	0%	0.1	0.6	0.8	−0.4	1.3	1.6	0.3	0.4	0.8
	FFF	20%	0.0	0.6	0.6	−0.2	0.7	1.5	0.1	0.8	0.7
	W3mmAL	21%	−0.4	2.3	0.6	−0.2	0.8	1.7	0.3	1.3	0.7
	FFF+WpatAL	34%	0.1	0.6	0.6	−0.2	0.5	1.6	0.1	0.8	0.7
	AD	51%	−0.1	0.6	0.9	−0.2	0.4	1.5	0.0	0.4	0.9
	AW	56%	0.0	0.4	0.7	0.2	0.7	1.8	0.1	0.5	0.8
*ABDOMEN (27 patients) (623 fractions)*	NI	0%	0.2	0.6	0.7	0.1	0.7	3.0	−0.1	0.6	0.9
	FFF	29%	0.0	0.3	0.7	0.2	0.8	3.3	−0.1	0.6	0.8
	W3mmAL	21%	0.0	0.8	0.6	0.1	1.2	3.4	0.0	0.9	0.8
	FFF+WpatAL	41%	−0.1	0.3	0.8	0.2	0.9	3.7	−0.1	0.5	0.9
	AD	51%	0.0	0.5	0.6	0.1	1.2	4.2	−0.1	0.4	0.8
	AW	58%	0.0	0.3	0.7	0.3	1.2	4.4	−0.1	0.5	0.8
*PROSTATE (41 patientes) (1508 fractions)*	NI	0%	0.4	0.4	0.5	−0.1	0.5	0.9	0.0	0.4	0.5
	FFF	14%	0.0	0.2	0.5	0.0	0.8	0.8	0.0	0.3	0.5
	W3mmAL	21%	−0.2	0.5	0.5	0.0	0.6	0.9	−0.2	0.6	0.5
	FFF+WpatAL	30%	0.0	0.2	0.6	0.0	0.8	0.9	−0.1	0.3	0.6
	AD	50%	0.0	0.1	0.5	0.0	0.3	1.0	−0.1	0.2	0.5
	AW	52%	0.0	0.1	0.6	0.0	0.3	1.0	0.0	0.2	0.6
*PELVIS (37 patients) (888 fractions)*	NI	0%	0.3	0.5	0.6	−0.1	0.5	0.8	−0.1	0.5	0.6
	FFF	26%	0.0	0.4	0.6	0.0	0.5	0.8	0.0	0.4	0.6
	W3mmAL	21%	0.0	0.6	0.6	0.0	0.7	0.8	0.1	0.6	0.6
	FFF+WpatAL	39%	0.0	0.3	0.6	0.0	0.4	0.8	0.0	0.3	0.7
	AD	51%	−0.1	0.3	0.6	0.0	0.4	0.9	0.0	0.4	0.6
	AW	56%	0.0	0.2	0.6	0.0	0.4	0.8	0.0	0.3	0.6
*EXTREMITIES (15 patients) (303 fractions)*	NI	0%	0.1	0.4	0.4	0.0	0.6	1.0	0.1	0.4	0.7
	FFF	40%	−0.2	0.4	0.7	0.1	0.5	1.0	0.2	0.5	0.7
	W3mmAL	22%	0.0	0.5	0.7	−0.1	0.6	1.1	0.1	0.6	0.7
	FFF+WpatAL	51%	−0.2	0.4	0.7	0.0	0.4	1.1	0.2	0.5	0.7
	AD	53%	0.1	0.2	0.9	0.0	0.4	1.3	0.1	0.5	0.8
	AW	64%	−0.1	0.3	0.7	0.0	0.3	1.0	−0.1	0.4	0.7

**Figure 4:**
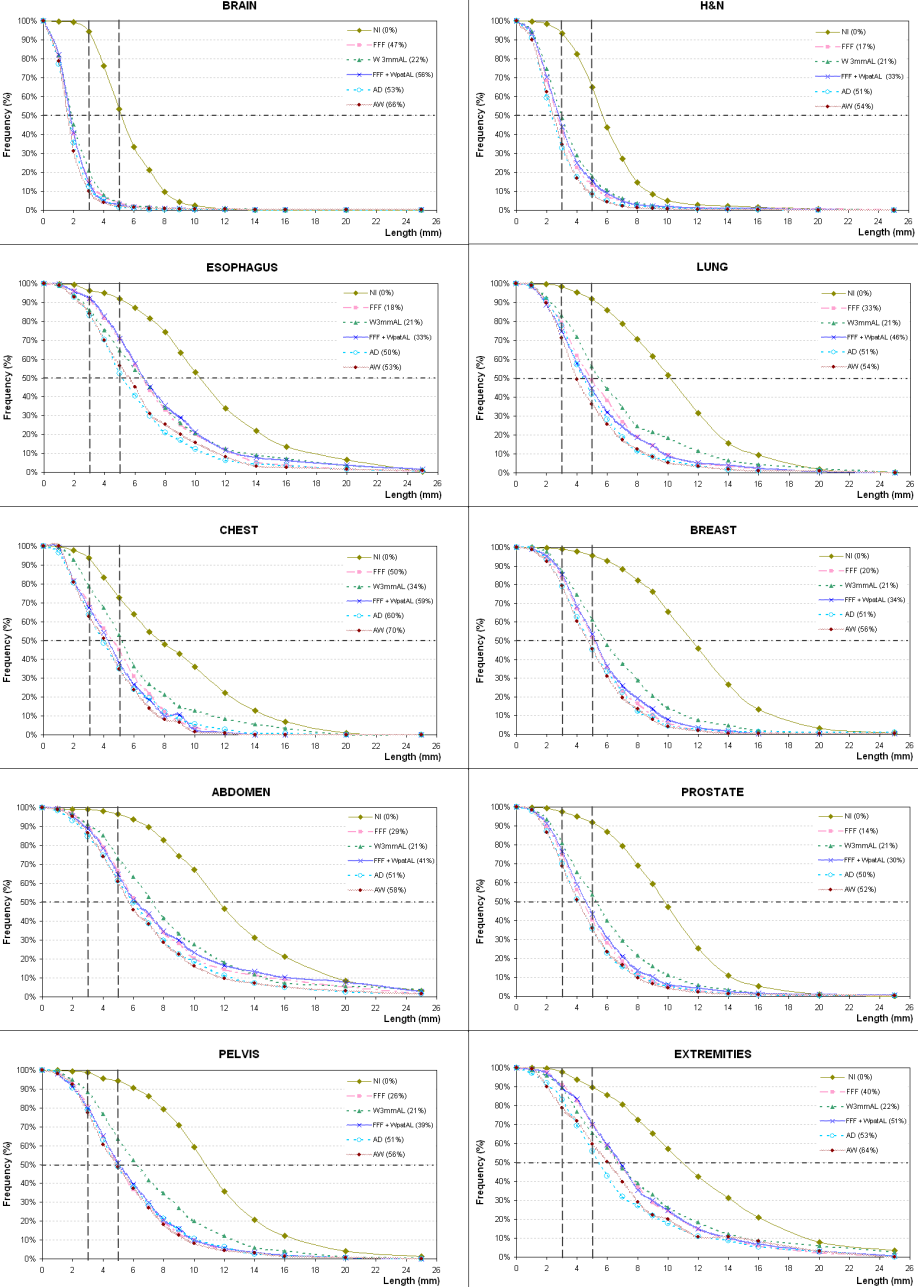
The frequency of the residual 3D setup error (truncated at 25 mm) is represented for all protocols and different anatomic sites. Nonimage‐guided fractions are included in the analysis. The IGf of each OVP is indicated in brackets.

The frequency distribution of 3D vector module of residual setup errors is plotted for treatment sessions that had no IG and for the different anatomic sites (Fig. 4). Overall, with the NI protocol more than 93% of delivered fractions were at least 3 mm off‐target in 3D space for all sites. However, the accuracy gained by increasing the IGf depends on the site treated. When the FFF protocol was used to reduce systematic error, only 15% and 41% of the subsequent corrected nonimaged fractions had residual errors of more than 3 mm, for brain and H&N, respectively; whereas, there were still more than 84% of the fractions that showed 3D residual setup error > 3 mm for anatomic sites where the PTV was a mobile target (esophagus, lung, abdomen, and breast) or was difficult to immobilize (extremity). For esophagus, lung, chest, and extremity patients, the AD and AW protocols (IGf ≥ 50%) got better results than any other ones, on reducing the number of non‐IG fractions with a 3D setup error > 5 mm (reduction 26%–10% compared with FFF), while the improvement on abdomen and prostate patients was 5%, and only 1% on brain and pelvis patients.

Any OVP, with IGf > 0%, achieves a margin reduction for all anatomic sites. The margins necessary to accommodate the residual uncertainties introduced by each protocol are 2–3 mm in all directions for brain patients, 3 or 5 mm for H&N patients, about 8 or 9 mm is still required in AP direction for the others anatomic sites, and up to 15 or 19 mm in SI direction for extremity and abdomen patients, respectively. In general, the W3mmAL protocol (IGf ≈ 21%) required the largest calculated treatment margins for all tumor sites, being even higher for esophagus patients when the FFF and FFF + WpatAL protocols were applied.

## IV. DISCUSSION

Our retrospective study of 389 patients (9418 fractions) is one of the largest in the literature for IGRT in multiple treatment sites. Although other authors^20^, ^21^ have made similar analyses, this study also investigates alternative OVPs in relation to the patient setup accuracy achieved. An adequate OVP would allow an improved utilization of the unit at the same time that dose escalation and OAR sparing through a reduction in the PTV remain possible in as safe way as daily IG.

### A. Daily setup correction

The systematic AP setup error is caused by couch sag^18^, ^22^, ^23^, ^24^ that occurs when patient is moved to treatment isocenter (inside the bore) from virtual isocenter located 70 cm outside. We obtained a mean systematic AP correction for NI protocol of 4.5 mm, whereas Zumsteg et al.^(25)^ found 0.56 mm for H&N patients treated in a linac equipped with a MV on‐board cone‐beam computed tomography (CBCT), both centers using the same thermoplastic mask. The magnitude of this error depends on the longitudinal position of the couch and the weight distribution on it. Therefore, the maximum vertical displacements are found for those treatment sites in which more table top is inside the tomotherapy unit's bore (i.e, breast, pelvis, prostate, and abdomen). In the case of the lower extremities, patients were simulated in supine feet first, so the table top held less weight and the AP displacement were similar to H&N or brain patients.

In SI direction, the systematic correction out of the bore has already been shown by other authors.^22^, ^26^, ^27^ This effect, may be due to several reasons: the couch top is being dragged into the bore by the patient's weight against the longitudinal drive mechanism;^(22)^ there is a mismatch between the CT laser and tomotherapy red lasers, and/or the MVCT slice resolution, as suggested by Hui et al.^(20)^ Woodford et al.^(11)^ observed that this scan resolution leads to large longitudinal shifts, independently of the combination of fusion technique and registration resolution selected.

### B. Off‐line protocols

The adequacy of the different OVPs can be assessed by the percentage of fractions which still have 3D vector residual setup error > 3 or 5 mm.

For H&N patients, the AW protocol resulted in smaller residual deviations than the FFF protocol (9% vs. 13% fractions had 3D vector > 5 mm) and CTV‐PTV margins, particularly in AP direction (3 mm instead of 5 mm). This result agrees with that reported by Vaandering et al.^(17)^ On the other hand, a similar NAL protocol (only 3 first fractions) studied by Houghton et al.^(22)^ got slightly worse results (29% sessions > 5 mm) than our FFF protocol. Other authors^17^, ^25^, ^28^ determine that daily IG should be used for patients with PTVs in close proximity to OARs or those with substantial residual errors due to daily random errors.

For brain patients, our study showed that the FFF protocol is accurate enough (85% of fractions were within a 3 mm margin) and efficient (IGf 47%), since the AW protocol would only achieve an improvement of 5% with IGf of 66%.

For esophagus patients the protocols AD and AW showed very similar results, but large residual errors were still observed, since more than 52% of fractions had residual 3D vector > 5 mm. This can be due to the difficulty of avoiding patient rotations (lower extremities were not immobilized), of achieving a good registration for such long PTVs, and/or possible patient's weight loss when they are treated with concomitant chemoradiotherapy. However, Chen et al.^(29)^ have not found any correlation between body habitus and daily setup errors. Despite the residual errors found, a substantial margin reduction is obtained if these protocols (with an IGf ≥ 50%) are applied. Nevertheless, Han et al.^(30)^ reported that, even with 60% IGf for esophageal cancer, 10% of the fractions had more than 10% decrease in the dose level covering 95% of the target.

For the treatment sites affected by respiratory motion (lung, chest, breast, and abdomen), the image registration process introduces itself an additional uncertainty because of MVCT blurring, as Smith et al.^(23)^ indicated. Since the MVCT rotation period (10 s) is slower than the respiratory period (typically 3–6 s), these images yield a tumor‐encompassing volume, whereas planning CT acquisitions are usually faster obtaining an almost static scenario. However, the residual setup errors cannot be justified only on the basis of respiratory motion, because it affects all image‐guided sessions in a similar way. In fact, all protocols required relatively small treatment margin expansions compared with the uncertainty introduced by tumor motion due to respiration, which is typically much larger in magnitude (AAPM Task Group 76^(31)^ reported 3.9–18.5 mm in SI direction for lung tumors) than residual systematic and random errors. Actually, Offerman et al.^(32)^ suggest that setup variation does not improve or degrade with repeated treatment setups for whole breast treatment, since no correlation between degree of daily shift and time course was found. For patients with boost or partial breast irradiation, Harris et al.^(33)^ showed that is possible to use the NI protocol when a PTV margin of 10 mm is applied, although high‐risk patients receiving simultaneous integrated boost with steep dose gradients may benefit from a margin reduction of 4 mm using a eNAL protocol, such as our AW protocol. However, Goddu et al.^(34)^ observed significant dose differences when 11 mm shifts in the anterolateral and 3 mm shifts in the posteromedial directions were simulated. For lung patients, Higgins et al.^(35)^ found that using first five‐day CBCT with a threshold of 3 mm produced worse residual setup error than even no IG. This protocol is similar to our OVP FFF, which presented better results than OVP W3mmAL, and even quite comparable to OVP FFF+WpatAL. For abdomen patients, a SI margin of 19 mm was needed for FFF and FFF+WpatAL protocols, where pancreatic cases were included and for which Li et al.^(36)^ reported pancreas movement up to 20 mm in LR and SI directions, so daily IG would be advisable.

For prostate and pelvis locations, all OVPs examined could reduce M(μ) and Σ(μ) to < 0.5 mm and 2 mm, respectively, with a relatively few numbers of IG sessions (Table 1). Again the FFF protocol seems to be a good trade‐off between MVCT workload and the correction of systematic errors. De Boer and Heijmen^(12)^ suggest that three days of imaging may be enough, although other authors concluded that four^(27)^ or ten^(24)^ image sessions are necessary to account for the systematic errors. Kupelian et al.^(26)^ suggest that daily imaging must be performed because residual errors are still significant at the 5 mm level, even with AD protocol. Our results showed that 40% of the fractions had a residual 3D vector > 5 mm with the FFF protocol, and the AD protocol only got an improvement of 5% (Fig. 4). Whichever OVPs are used, the margins will be larger than those required for daily image correction, which will result in higher toxicity (e.g., hematologic toxicity due to higher volume irradiation of pelvic bone marrow, as Chen et al.^(37)^ have shown for anal cancer).

For extremity patients, none of the OVPs achieved to reduce Σ(μ) errors in SI direction. If FFF or AW protocol was used, the calculated margins were 10–9 mm in LR and AP direction, and 14–13 m in SI direction, respectively. Dickie et al.^(38)^ quantified a uniform margin of 5 mm for lower extremity soft tissue sarcoma, which are lower than ours probably because of different immobilization used.

For all sites analyzed, the margins to CTV‐to‐PTV agree with the actual margins used in our clinical practice. On the one hand, this implies that margins could be reduced if on‐line protocol is used. On the other hand, it would not be necessary to have two plans, one for IGRT and one for non‐IGRT sessions, as other authors refer.^(39)^

Our model couch (Hi·Art Legacy Couch) along with software version 4.1 only allows to apply to vertical corrections when MVCT scan is not performed, so the reducing of image workload does not result in a great saving of time. Therefore, we consider that the benefit of the daily image for the patient is greater than the time saved achieved. However, the philosophy of the OVPs could be applied on days of the important treatment delays, such as machine interruptions and/or breakdowns, to ensure that all patients could be treated.

Despite daily MVCT scans involving an additional dose of 0.01 Gy per fraction^(40)^ for H&N patients, Duma et al.^(6)^ and Schwarz et al.^(39)^ have reported that daily IGRT has the smallest increase in dose to spinal cord when it is compared with other nonimage‐guided scenarios. In addition, Smith et al.^(23)^ showed that MVCT doses were lower than in other imaging modalities, such as CBCT, for which the IGf may be the greater concern for reducing imaging dose.

## V. CONCLUSIONS

This study presents a comprehensive set of data for ten anatomic sites throughout the patient's body. The setup errors and the feasibility of the different OVPs were site‐specific and were related with the immobilization devices used. The possibility to apply some of the OVPs has been shown for some treatment sites, such as FFF protocol for brain patients, or even for lung and breast patients, provided that the respiration‐induced motion has been considered during the initial margin construction from daily IG. However, we think that daily IG should be used for those locations where substantial anatomic changes can occur through the RT course, such as H&N, esophagus, prostate or abdomen patients, or when the immobilization is difficult, such as extremity patients, because even protocols with IGf ≥ 50% have setup errors of > 5 mm in 50% of the fractions, which could be unacceptable. Additionally, in our department the workload would not be significantly reduced due to the fact that it is very time consuming to perform the setup corrections with our current model of couch.
